# Additive Effects of Environmental Enrichment and Ketamine on Neuropathic Pain Relief by Reducing Glutamatergic Activation in Spinal Cord Injury in Rats

**DOI:** 10.3389/fnins.2021.635187

**Published:** 2021-03-22

**Authors:** W. L. Tai, L. Sun, H. Li, P. Gu, E. A. Joosten, C. W. Cheung

**Affiliations:** ^1^Laboratory and Clinical Research Institute for Pain, Department of Anesthesiology, Queen Mary Hospital, The University of Hong Kong, Hong Kong, China; ^2^The First Rehabilitation Hospital of Shanghai, Brain and Spinal Cord Innovation Research Center, Advanced Institute of Translational Medicine, Tongji University School of Medicine, Shanghai, China; ^3^Department of Anesthesiology and Pain Management, University Pain Centre Maastricht (UPCM), Maastricht University Medical Centre, Maastricht, Netherlands; ^4^Department of Translational Neuroscience, School for Mental Health and Neuroscience, Maastricht University, Maastricht, Netherlands

**Keywords:** environmental enrichment, ketamine, neuropathic pain, neuroplasticity, neuroprotection, NMDA receptor, spinal cord injury

## Abstract

Spinal cord injury (SCI) impairs mobility and often results in complications like intractable neuropathic pain. A multi-approach management of this chronic pain condition has been encouraged, but little has been explored of the field. Here, we focus on the effect and underlying mechanism of environmental enrichment (EE), which promotes voluntary social and physical activities, combined with a clinical analgesic, ketamine, on SCI-induced neuropathic pain as well as motor dysfunction. We performed T13 spinal hemisection in rats, which induced unilateral motor impairment and neuropathic pain-like behaviors in the hindlimb. Treatment regimen started a week after SCI, which consists of ketamine administration (30 mg kg^–1^ day^–1^; intramuscular) for 10 days, or EE housing for 20 days, or their combination. Paw withdrawal response to mechanical and thermal stimuli, motor function, burrowing behaviors, and body weight was monitored. Spinal segments at T13 lesion and L4–L6 were collected for histopathological and protein analyses. The joint treatment of EE and ketamine provided greater relief of pain-like behaviors and locomotor recovery than did either paradigm alone. These improvements were associated with reduced cavitation area, astrogliosis, and perilesional phosphorylation of glutamate *N*-methyl-D-aspartate receptor (NMDAR). Concurrently, lumbar spinal analysis of NMDAR-linked excitatory markers in hypersensitization showed reduced activation of NMDAR, mitogen-activated protein kinase (MAPK) family, nuclear factor (NF)-κB, interleukin (IL)-1β signaling, and restored excitatory amino acid transporter 2 level. Our data support a better therapeutic efficacy of the combination, EE, and ketamine, in the attenuation of neuropathic pain and motor recovery by reducing spinal glutamatergic activation, signifying a potential multifaceted neurorehabilitation strategy to improve SCI patient outcome.

## Introduction

Chronic neuropathic pain develops in approximately 65% of people following spinal cord injury (SCI), severely compromising patient’s life on top of motor impairment (Siddall et al., 2003; Duenas et al., 2016). Despite rigorous research effort, available treatments remain limited and undesirable (Ramer et al., 2014). Since chronic pain and neurodegeneration are strongly linked (Costigan et al., 2009), a multitudinal intervention targeting both complications may result in complementary improvement of sensory and motor functions in SCI.

Environmental enrichment (EE) is a preclinical model of rehabilitation that facilitates voluntary motor, sensory, and cognition activities by provision of a stimulating environment, avoiding the risk of exercise overload. It is well-documented to enhance neurogenesis and locomotion, enabling its translational use in neurorehabilitation unit (Janssen et al., 2012; Tai et al., 2018b), but its role in post-SCI recovery remains largely unknown. A few preclinical studies demonstrated that SCI animals housed in enriched environment have shown reduced lesion volume and improved sensorimotor recovery (Berrocal et al., 2007; Koopmans et al., 2012). Moreover, EE has been proven beneficial in attenuating allodynia and hyperalgesia in both neuropathic and inflammatory pain models (Gabriel et al., 2009; Stagg et al., 2011). However, such analgesic effects remain partial (Berrocal et al., 2007; Koopmans et al., 2012). In fact, studies of traumatic brain injury have suggested that combination of EE with selective pharmacotherapies can confer added benefits (de la Tremblaye et al., 2019).

Ketamine, a classic anesthetic, provides strong analgesia by blocking the glutamate *N*-methyl-D-aspartate receptors (NMDARs) (Bell, 2017). In the recent decade, low dosage of ketamine has shown distinctive analgesic efficacy in neuropathic pain, which warrants a favorable safety profile, escalating its clinical use (Schwartzman et al., 2009; Amr, 2010; Pourmand et al., 2017). Remarkably, ketamine continues to produce neuropathic pain-relief when its effective drug level subsided (Sleigh et al., 2014). It is possible that subanesthetic ketamine may be an effective adjunct to compensate the limited analgesic effects of EE.

The combination of EE and ketamine may provide additive therapeutic benefit by targeting the glutamatergic system, which is essential in neuroplasticity and pain (Kentner et al., 2016; Bell, 2017). Dysregulation of glutamate transmission (i.e., glutamate excitotoxicity and NMDAR overexcitation) contributes to central sensitization and neurodegeneration in SCI (Paoletti et al., 2013). EE was shown to reduce spinal NMDAR phosphorylation following multiple sclerosis (Benson et al., 2015), and ketamine was found to restrict excitotoxicity and confer neuroprotection by NMDAR inhibition (Bell, 2017). Among the NMDAR subtypes, a crucial role of the upregulated NR2B-containing NMDAR has been highlighted in chronic pain, but not in acute nor physiological pain (Zhuo, 2009). NR2B-NMDAR activation has been specifically related to excitotoxicity and neuronal cell death (Hardingham and Bading, 2010). The current study aimed to investigate the efficacy of combination of EE and ketamine in attenuating SCI-induced neuropathic pain and motor defects as well as on NR2B-NMDAR activity.

## Materials and Methods

### Animal

Adult male Sprague–Dawley rats (250–300 g) were kept individually in plastic cages with floor covered with soft bedding at room temperature and maintained on a light/dark cycle of 12-h day/night. Food and water were provided *ad libitum*. Animal experiments were conducted according to the US National Institute of Health Guide for the Care and Use of Laboratory Animals and were approved by the Committee on the Use of Live Animals in Teaching and Research from The University of Hong Kong (Project #3498-14).

### Experimental Design

**FIGURE 1:**
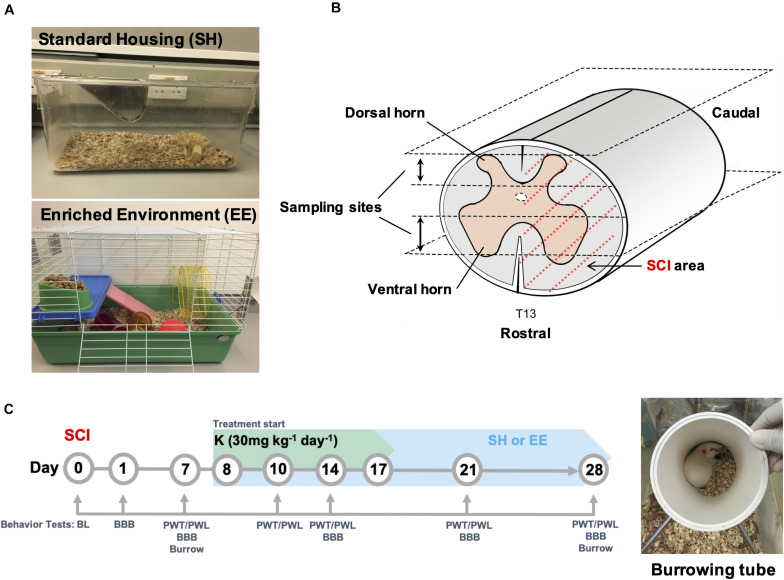
Experimental design. **(A)** Overview of the standard and enriched environment (EE) housing. **(B)** Schematic demonstration of T13 spinal cord hemisected area as covered by dotted red line. Upper and lower sampling sites represent the dorsal and ventral spinal columns respectively where tissue sections for histopathological analysis were collected. **(C)** Time course of experimental treatments and behavioral tests. A tube used in the burrowing test is shown in the image. BL, Baseline; BBB, Basso, Beattie, and Bresnahan locomotor test; PWT, paw withdrawal threshold; PWL, paw withdrawal latency.

Animals were randomly assigned to the following: sham, SCI control (SCI group), SCI plus ketamine (K group), SCI plus EE (EE group), and SCI plus EE, and ketamine (EEK group). Before surgery, standard housing was applied to all animals that they were housed two *per* cage in a conventional 1291H rat cage [42.5 cm (L) × 26.6 cm (W) × 18.5 cm (H)] (Lab Animal Unit, The University of Hong Kong, Hong Kong, China) with nesting materials (sawdust) only. After surgery, all animals were housed individually in standard housing condition for a week. Then rats in the SCI and K groups were kept in the same setting onward, while rats in the EE and EEK groups were housed three *per* EE cage (Figure 1).

Behavioral tests were performed under blinded conditions preoperatively [baseline (BL)] and on postoperation day (POD) 1, 7, 10, 14, 21, and 28 based on test type (Figure 1C). Of note, for the K and EEK groups, behavior tests were performed before ketamine injection when the test day overlapped with ketamine administration day, to avoid any immediate influence of ketamine injection.

### Environmental Enrichment Housing

The EE housing is adapted from a previous study (Gabriel et al., 2009) and modified to comply with guidelines of the Lab Animal Unit of The University of Hong Kong. The EE setup consists of a paint-coated metallic wire cage [69 cm (L) × 45 cm (W) × 43 cm (H)] with various objects inside, such as a running wheel [23 cm (diameter) × 9 cm (platform width) × 26 cm (total height)], a crawl ball, climbing frames, a tunnel, a jingle ball, and additional nesting material (Lab Animal Unit) (Figure 1A). Objects in the cage were renewed once every week. Water and food were placed at the opposite end of the cage. With this setup, animals had extra space to explore with moderate, voluntary exercise by walking back and forth between the water and food and by using the added attributes.

### Spinal Cord Injury Model

T13 hemisection of the spinal cord was performed according to an established protocol, a moderate SCI model that allows recovery of basic reflex around POD 7 for pain behavior assessments (Christensen et al., 1996). In brief, under general anesthesia with isoflurane (2% for induction and 1% for maintenance in 70% N_2_O/30% O_2_), rats underwent left hemi-laminectomy at T13 after hair removal and sterilization of the surgery area with 75% ethanol and Betadine. A small slit was made in the dura, and the spinal cord was hemisected with fine iridectomy scissors (FST, Linton, United Kingdom), leaving intact the dorsal vessel and its major vascular branches. Sham-operated animal underwent the same surgical process without the hemisection of the spinal cord.

According to the guidelines provided by the Committee on the Use of Live Animals in Teaching and Research, the depth of anesthesia was monitored every 15 min during surgery by observation of the following: lack of pedal withdrawal to painful stimulus, heart rate (300–500 beats min^–1^), respiration rate (70–110 breath min^–1^), saturated pulse oxygenation (i.e., mucous membrane color of mouth, pink or pale pink), and body temperature (37.5–38.5°C).

### Intramuscular Administration of Ketamine

Ketamine (Sigma, St. Louis, MO, United States) was dissolved in saline and injected intramuscularly at a dose of 30 mg kg^–1^ day^–1^ for 10 consecutive days starting from POD 8 (Figure 1C). Drug dosage was adapted from a previous study to obtain a low dose within the subanesthetic range of ketamine (Goldberg et al., 2005; Amr, 2010).

### Mechanical Allodynia and Thermal Hyperalgesia

Mechanical and thermal thresholds were evaluated before surgery and on POD 7, 10, 14, 21, and 28 (Figure 1C) as described previously (Sun et al., 2018). In brief, animals were placed individually in plexiglass boxes on a stainless steel mesh floor (for mechanical test) or on a transparent glass surface (for thermal test) for at least 15 min to habituate on the test day. The degree of mechanical allodynia was evaluated by quantifying paw withdrawal threshold (PWT) of the ipsilateral hindpaw in response to mechanical stimulation (innocuous) using a calibrated electronic von Frey filament anesthesiometer (IITC Life Science, Woodland Hills, CA, United States) with blunted Von Frey filaments. Animals’ sensitivity to noxious heat was evaluated using the plantar test, carried out with a paw algesia meter (IITC). A focused, adjustable, radiant heat light source beneath a glass floor was applied at the plantar surface of the ipsilateral hindpaw. Noxious (50% intensity, cutoff time 20 s) heat stimulus was applied to determine the paw withdrawal latency (PWL). Three individual readings were taken in an animal with an interval of at least 5 min and averaged.

### Motor Function Assessment

Before injury and on POD 1, 7, 14, 21, and 28, rats were examined for motor function in an open-field test space using the Basso, Beattie, and Bresnahan (BBB) locomotor rating scale (Basso et al., 1995; Figure 1C). In brief, the BBB scale ranges from 0 (no hindlimb movement) to 21 (normal movement, including coordinated gait with parallel paw placement). Scores from 1 to 7 indicate the early phase of recovery with return of slight to extensive movements in the three joints (hip, knee, and ankle). Scores from 8 to 13 represent the intermediate phase of recovery where the return of paw placement and coordinated movements with the forelimbs is observed. Scores from 14 to 21 show the late phase of recovery with reappearance of toe clearance during stepping, predominant paw position, trunk stability, and tail position. Only the scores of the hindlimb on the hemisected side (ipsilateral side) were shown since there were no observable differences in locomotion of the contralateral side.

### Burrowing Assay

Rodents are burrowing mammals, and their burrowing ability is innate, natural, and highly conserved. Therefore, burrowing assay is considered as an effective measure of overall animal well-being (Deacon, 2006) and an indirect measure of pain behaviors, because it does not involve stimulus-evoked pain response (e.g., PWT and PWL) (Andrews et al., 2012; Rutten et al., 2014). The construct of this paradigm for current study is that SCI-induced neuropathic pain and motor impairment would affect a rat’s motivation and ability to burrow. Burrowing test was carried out as previously described (Deacon, 2006) with slight modifications before surgery and on POD 7 and 28 (Figure 1C). A burrowing tube (23.5 cm long × 10 cm diameter, Figure 1C) was raised by two metal stands at the open end to approximately 60 mm higher than the closed end (constructed in-house at The University of Hong Kong, Hong Kong, China). The tube was filled with 1-kg pea shingle gravel with a diameter of 2 to 4 mm (JHC, Hong Kong, China) and placed in a test cage with slightly sprinkled fresh bedding at the start of each test.

Specifically, all rats received three training sessions as suggested by previous studies (Andrews et al., 2012; Rutten et al., 2014) on three consecutive days before the burrowing performance test. On training day 1, rats were placed in cages in pairs for 1 h of habituation. Then, an empty tube was placed into the cage for 1 h to allow familiarization. On days 2 and 3, the procedure was repeated but with a burrow filled with 1 kg of gravel. Following the third day of training, rats that demonstrated a tendency to burrow (∼90% of those tested) underwent the same procedure as on the previous day but were tested individually to determine each rat’s baseline level of burrowing. The amount of gravel left in the burrow at the end of each test session was weighted and recorded as a measure of the burrowing behavior. Animals with burrowing baselines <200 g were excluded from the experiment (<10%).

### Tissue Preparation

At the end of the experiments (POD 28), the spinal cords were harvested from the experimental animals after euthanization with sodium pentobarbital. The ipsilateral spinal cord segments of L4–L6 were removed, snap-frozen in liquid nitrogen, and stored at −80°C until further protein extractions. For immunofluorescence analysis, spinal segment of T12–T13 centered at the lesion site was dissected from rats perfused with 4% paraformaldehyde (PFA) and then fixed in 4% PFA and dehydrated in 30% sucrose. Specimens were then cryoprotected in optimal cutting temperature gel. Twenty-four serial horizontal cryosections (10 μm thick) were cut from each gray matter-containing spinal column on the dorsal and ventral sides (Figure 1B), using a microtome cryostat (Leica Microsystem, Wetzlar, Germany). Once every four sections in a series of 24 sections was chosen to process for paralleled comparison in histochemistry studies.

### Histopathology and Quantification

Sections were subjected to Nissl staining for assessment of tissue cavity. The pattern of Nissl-stained neurons allowed the identification of the spinal dorsal and ventral columns as well as gray matter preservation. The sections were stained in 0.1% toluidine blue solution for 3 min and then rinsed briefly in distilled water followed by differentiation in 95% ethyl alcohol for 5 min. The boundary of cavitation area was bordered by the spared tissue. The resulting area was calculated using ImageJ (National Institutes of Health, Bethesda, MD, United States) by converting the pixels into millimeters. A total of five tissue sections centered at the cavitation were analyzed in each animal.

### Immunofluorescence and Quantification

Sections were subjected to wash with phosphate-buffered saline for three times, 5 min each time, before blocking of non-specific binding. To assess astrocytic reactivity, sections were stained with astrocyte marker, glial fibrillary acidic protein (GFAP). For double staining, sections were incubated with primary antibodies of phosphorylated (p)-NR2B and neuron marker, NeuN. For triple-labeling, sections were incubated in p-NR2B antibody with microglia marker, Iba1, and GFAP, or with Iba1 and NeuN, respectively, at 4°C overnight. Details of primary antibodies are provided in Table 1. Subsequently, sections were incubated with donkey anti-rabbit, mouse, or goat secondary antibodies conjugated with Alexa Fluor 488, 568, and 647, respectively (1:1,000, Abcam, Cambridge, United Kingdom). The slides were cover-slipped in mounting medium containing DAPI and visualized by Zeiss LSM 780 confocal microscope (Zeiss, Jena, Germany).

**TABLE 1:** Primary antibodies for immunofluorescence.

Antibody	Dilution	Vendor
Rabbit anti-NMDAR2B, phospho Tyr 1472	1:100	Merck Millipore, Darmstadt, Germany
Goat anti-Iba-1	1:50	Novus Biologicals, Littleton, CO, United States
Mouse anti-NeuN	1:300	Abcam, Cambridge, United Kingdom
Mouse anti-GFAP	1:250	

Astrocytic immunoreactivity was semi-quantified by measurement of immunodensity in an area of 1,800 × 800 μm centered at the hemisection epicenter using ImageJ (National Institutes of Health, Bethesda, MD, United States) as previously described (Sun et al., 2018). The relative immunodensity was normalized to the corresponding area in the sham group. For quantification of double-labeled cells, NeuN(+) and NeuN(+)/p-NR2B(+) cells were counted in the area of 500 μm caudal to the hemisection epicenter. Data were calculated as a percentage of p-NR2B-positive neurons over the total number of neurons in the dedicated area. A total of five spinal sections were analyzed in each animal.

### Western Blotting

Frozen spinal cord samples were homogenized (Polytron Kinematica, Lucerne, Switzerland) in 1 ml of ice-cold lysis buffer with protease inhibitor cocktails (Sigma, St. Louis, MO, United States). Clear supernatants were collected after centrifugation, and protein concentrations were determined by the Bradford method (Bio-Rad, Hercules, CA, United States). Protein extracts were subjected to electrophoresis in 10% sodium dodecyl sulfate–polyacrylamide gel electrophoresis (SDS-PAGE) gels and subsequently transferred to polyvinylidene fluoride membranes (Bio-Rad). After being blocked with 5% non-fat milk for 1 h, membranes were probed with primary antibodies listed in Table 2 overnight at 4°C in incubation buffers. After being washed with Tris-buffered saline-Tween 20, the membranes were incubated for 2 h at room temperature in with proper secondary antibodies horseradish peroxidase (HRP)-linked anti-rabbit or anti-mouse IgG (Cell Signaling Technology, Danvers, MA, United States). Protein expressions were detected on films by enhanced chemiluminescence (Bio-Rad). The relative optical density of all bands was determined by quantifying the scanned image with ImageJ software (National Institutes of Health).

**TABLE 2:** Primary antibodies for western blotting.

Antibody	Dilution	Vendor
Rabbit anti-phospho-ERK1/2 (p-ERK1/2)	1:1,000	Cell Signaling Technology, Danvers, MA, United States
Rabbit anti-ERK1/2	1:4,000	
Rabbit anti-phospho-p38 (p-p38)	1:1,000	
Rabbit anti-p38	1:4,000	
Rabbit anti-phospho-JNK (p-JNK)	1:1,000	
Rabbit anti-JNK	1:4,000	
Rabbit anti-phospho-NF-κB p65, Ser536 (p-NF-κB)	1:1,000	
Rabbit anti-NF-κB p65	1:4,000	
Monoclonal anti-GAPDH	1:4,000	
Rabbit anti-phospho-NMDAR2B Tyr 1472 (p-NR2B)	1:1,000	Merck Millipore, Darmstadt, Germany
Rabbit anti-NMDAR2B (NR2B)	1:3,000	
Rabbit anti-EAAT2	1:3,000	Abcam, Cambridge, United Kingdom
Rabbit anti-IL-1β	1:1,000	

### Statistical Analysis

All numerical values were stated as mean ± standard deviation (SD) and analyzed using GraphPad Prism 6 software (GraphPad Software Inc., La Jolla, CA, United States). Sample size was estimated based on previous similar studies (de la Tremblaye et al., 2017; Shi et al., 2018) and our experience (Tai et al., 2018a). Temporal behavior study and animal body weight were analyzed using two-way analysis of variance (ANOVA) with repeated measures followed by Tukey’s *post hoc* test. Area under the curve (AUC) and comparison of data from the histological study and western blot were analyzed with one-way ANOVA followed by Tukey’s *post hoc* test. *P* < 0.05 was considered statistically significant.

## Results

### Early Onset and Continuous Relief of Mechanical and Thermal Hypersensitivities by Joint Treatment of Environmental Enrichment and Ketamine in Spinal Cord Injury Rats

**FIGURE 2:**
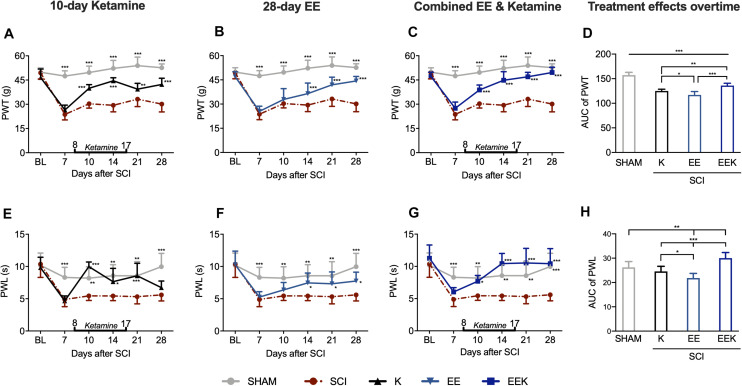
Environmental enrichment (EE) and ketamine (K) reduced nociceptive responses in hindpaws after spinal cord injury (SCI). **(A–C)** EE (*n* = 14), K (*n* = 8), and their combination EEK (*n* = 9) treatments alleviated SCI-induced mechanical allodynia [paw withdrawal threshold (PWT)]. *n* = 6 in sham and *n* = 8 in the SCI group. Two-way repeated-measures ANOVA (effect vs. group × time interaction) followed by Tukey’s *post hoc* test. *F*_*ketamine*_(10,95) = 13.78, *F*_*ee*_(10,125) = 13.65, *F*_*eek*_(10,100) = 16.84. *P < 0.05, **P < 0.01, ***P < 0.001 vs. SCI. **(D)** Comparison between treatment effects over time by measuring area under the curve (AUC) demonstrated a higher efficacy of the joint treatment than individual ones in combating allodynia. One-way ANOVA (effect vs. group) followed by Tukey’s *post hoc* test. *F*(3,33) = 78.27. *P < 0.05, **P < 0.01, ***P < 0.001. **(E–G)** SCI-induced thermal hyperalgesia [paw withdrawal latency (PWL)] was reduced by either ketamine (*n* = 8) or EE (*n* = 14) and was reversed by the EEK group (*n* = 9). *n* = 6 in sham and *n* = 7 in the SCI group. Two-way repeated-measures ANOVA (effect vs. group × time interaction) followed by Tukey’s *post hoc* test. *F*_*ketamine*_(10,90) = 5.665, *F*_*ee*_(10,120) = 2.166, *F*_*eek*_(10,95) = 4.218. *P < 0.05, **P < 0.01, ***P < 0.001 vs. SCI. **(H)** Between-treatment comparison over time (AUC) demonstrated an added benefit of the combined treatment EEK in hyperalgesia relief. AUC is computed from timepoints day 10 to 28, which are after treatment started. One-way ANOVA (effect vs. group) followed by Tukey’s *post hoc* test. *F*(3,33) = 27.72. *P < 0.05, **P < 0.01, ***P < 0.001. Data are presented as mean ± standard deviation (SD). Double-end line in bold indicates the 10-day period of ketamine administration (day 8 to 17). Sham, sham-operated group; BL, baseline.

Rats developed mechanical allodynia after SCI that persisted for at least 28 days, which was demonstrated by significantly lower PWT in the ipsilateral hindpaws of SCI rats than that in sham (all *P* < 0.001, *n* = 8, Figures 2A–C). In contrast to the SCI group, 10 days’ injection of subanesthetic ketamine (30 mg kg^–1^ day^–1^, intramuscular; Figure 2A) significantly increased PWT by 2 days after initial injection. Although a slight drop in PWT was seen after ketamine discontinued on POD 17, the remaining drug effect continued to relieve pain-like behavior till experiment end point (POD 10 to 28; all *P* < 0.01). EE housing took effect later than ketamine did but in a progressive manner (Figure 2B). It first increased PWT by a week after housing began and continued to further elevate PWT progressively (POD 14 to 28; all *P* < 0.001). In comparison, the combination of ketamine and EE markedly increased PWT starting from POD 10 (all *P* < 0.001; Figure 2C) and reversed the threshold to basal level by POD 28 (*P* = 0.438, EEK: 49.629 ± 3.270 g vs. sham: 52.645 ± 2.332 g). To evaluate differences in the therapeutic effects between treatment groups overtime, we compared the AUC of PWT of treated groups, which is a measure of both magnitude and duration. Over the experiment time course, the joint treatment conferred significantly better relief of allodynia than that by ketamine or EE alone (*P* < 0.01; Figure 2D).

Thermal hyperalgesia manifested as significant decrease of ipsilateral PWL in response to noxious heat stimulus after SCI compared with sham (POD 7 to 28, all *P* < 0.01; Figures 2E–G). After 2 days of ketamine injection (Figure 2E), PWL spiked as observed on POD 10 (*P* < 0.001, K: 9.987 ± 0.702 s vs. SCI: 5.442 ± 0.713 s) and returned to the basal level from POD 10 to 21 (all *P* > 0.05 vs. sham). After ketamine cessation (POD 17), such analgesia lasted at least 4 days (POD 21: *P* < 0.001) but was unstable and ceased by 11 days post treatment (POD 28: *P* = 0.660). On the other hand, EE gradually increased PWL, which reached a significant level by POD 14 (*P* < 0.05, EE: 7.452 ± 1.535 s vs. SCI: 5.432 ± 0.724 s). This effect was maintained throughout the experiment where PWL returned to the basal level from POD 14 to 21 (all *P* > 0.05 vs. sham, Figure 2F). In contrast, combined regimen EEK took effect early and reversed hyperalgesia-like behavior by 6 days after treatment commenced and returned PWL to the basal level from POD 10 to 28 (all *P* > 0.05 vs. sham). The observed significant analgesia lasted through the experiment time course (POD 14 to 28: all *P* < 0.001 vs. SCI, Figure 2G). Overtime, the combination treatment demonstrated a strong efficacy against thermal hyperalgesia, surpassing the effects of ketamine or EE significantly (*P* < 0.001; Figure 2H).

### Environmental Enrichment Combined With Ketamine Improves Functional Recovery and Global Well-Being of Spinal Cord Injury Rats

**FIGURE 3:**
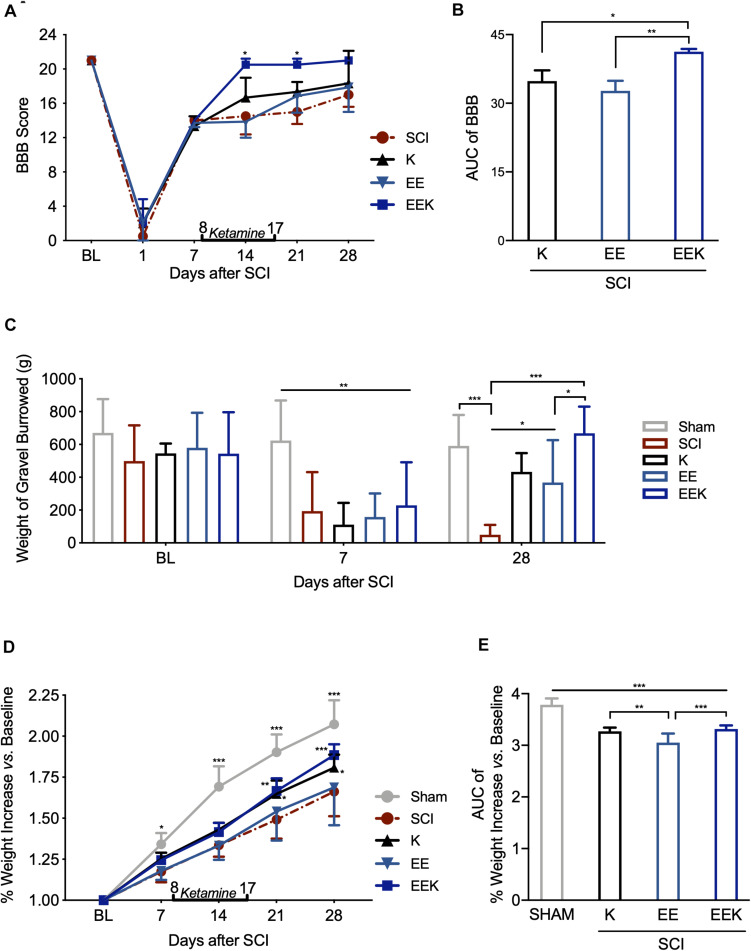
Environmental enrichment (EE) and ketamine (K) promoted motor recovery and general well-being after spinal cord injury (SCI). The combined treatments of EE and K (EEK) restored the SCI-impaired **(A,B)** motor function, *n* = 3 in SCI, *n* = 3 in K, *n* = 4 in EE, and *n* = 4 in EEK groups [score of Basso, Beattie, and Bresnahan locomotor test (BBB)]; **(C)** burrowing ability, *n* = 6 in sham, *n* = 6 in SCI, *n* = 5 in K, *n* = 8 in EE, and *n* = 8 in EEK (burrowed gravel weight); and **(D–E)** body weight, *n* = 6 in sham, *n* = 8 in SCI, *n* = 8 in K, *n* = 14 in EE, and *n* = 9 in EEK. **(A,C,D)** Two-way repeated-measures ANOVA (effect vs. group × time interaction) followed by Tukey’s *post hoc* test; **(A)**: *F*(15,50) = 1.985, **(C)**: *F*(8,56) = 5.293, **(D)**: *F*(16,160) = 8.196. *P < 0.05, **P < 0.01, ***P < 0.001. **(B,E)** Area under the curve (AUC) is computed from timepoints day 14 to 28, which are after treatment started. One-way ANOVA (effect vs. group) followed by Tukey’s *post hoc* test. **(B)**: *F*(2,9) = 12.65, **(E)**: *F*(3,33) = 44.99. *P < 0.05, **P < 0.01, ***P < 0.001. Data are presented as mean ± standard deviation (SD). Double-end line in bold indicates the 10-day period of ketamine administration (day 8 to 17). Sham, sham-operated group; BL, baseline.

Rats subjected to SCI surgery lost ipsilateral hindlimb motor function immediately emerging from anesthesia. Early-to-intermediate motor recovery phase was observed during the first week after surgery demonstrated by elevated BBB score from POD 1 to 7 (Figure 3A). This agreed with previous studies that used the same spinal cord hemisection model as in this study, which allows return of intermediate motor reflex for reliable testing of pain behaviors (Christensen et al., 1996; Coronel et al., 2011). We observed that only the EEK group achieved full motor function recovery while others remained at the intermediate-to-late phase (*P* < 0.05, *n* = 8, EEK: 20.500 ± 0.707 vs. SCI: 14.5 ± 2.121). Over the time course, the combined treatment showed a significantly better effect than that of either K (*P* < 0.05) or EE (*P* < 0.01) in recovering locomotor after SCI (Figure 3B).

As a global assessment of sensorimotor function and non-stimulus-evoked pain response, animal’s burrowing behavior was observed at BL, before and after treatment (POD 7 and 28, respectively). SCI markedly reduced burrowed weight compared with sham, indicating burrowing behavioral deficit after injury (POD 7, all *P* < 0.01, Figure 3C). Ketamine or EE significantly increased the amount of gravel displaced (POD 28, both *P* < 0.05), while their combination restored the burrowed amount to the basal level (POD 28, *P* = 0.995, EEK: 667.625 ± 163.095 g vs. sham: 590.833 ± 188.337 g). Of note, the EEK group burrowed a greater amount than the EE group (*P* = 0.030).

In addition to burrowing behavior, animal’s body weight was also recorded as an evaluation of general well-being. Body weight of animals in all groups increased gradually over the experiment course, but the SCI group grew at a significantly lower rate than the sham group (all *P* < 0.5, Figure 3D). No significant difference was detected between the EE and SCI groups. In contrast, from 21 days after SCI, weight growth in both groups K (1.808 ± 0.079%, *P* = 0.02) and EEK (1.884 ± 0.066%, *P* < 0.01) was significantly greater than that in the SCI group (1.662 ± 0.149%). The weight increase overtime in the EEK and K groups was greater than that of the EE group (*P* < 0.001; Figure 3E).

### Environmental Enrichment and Ketamine Reduce Lesion Size and Gliosis in the Spinal Cord After Injury

**FIGURE 4:**
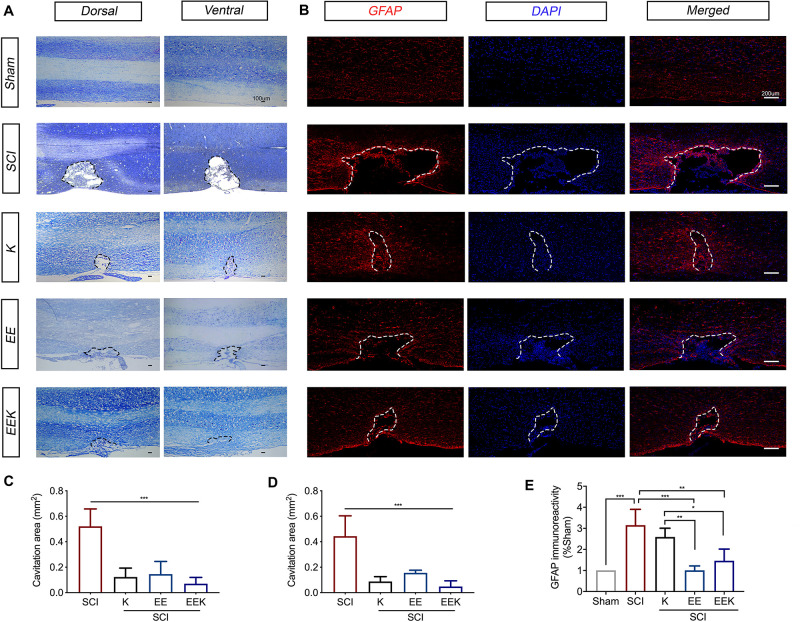
Environmental enrichment (EE), ketamine (K), and their combination (EEK) promoted wound heal and reduced gliosis following spinal cord injury (SCI). Representative sections of the **(A)** Nissl-stained dorsal and ventral spinal cord were from the same animal in each group to demonstrate an overview of the spinal cord hemisected site. The dorsal spinal cord can be identified by the dense pattern of small neurons relative to the scattered pattern of large neurons in the ventral. *n* = 5/group. **(B)** Representative images of astrocytic immunoreactivity in the spinal dorsal column. Quantification of Nissl-stained cavitation area in the **(C)** dorsal and **(D)** ventral spinal columns, as well as **(E)** glial fibrillary acidic protein (GFAP)-stained astrocytic immunoreactivity demonstrated reduced cavitation area and GFAP reactivity by all treatment groups. *n* = 3 in sham, *n* = 3 in SCI, *n* = 4 in K, *n* = 3 in EE, and *n* = 4 in EEK. One-way ANOVA (effect vs. group) followed by Tukey’s *post hoc* test; **(C)**: *F*(3,16) = 23.82, **(D)**: *F*(3,16) = 21.77, **(E)**: *F*(4,12) = 13.66. *P < 0.05, **P < 0.01, ***P < 0.001. Data are presented as mean ± standard deviation (SD). Cavitation area is bordered by dotted lines.

To assess the effect of EE and ketamine on SCI lesion size and astrogliosis, we performed Nissl staining and immunostaining for GFAP, respectively, on the longitudinal section of the spinal cord centering at the injury site. Nissl-stained spinal sections identified the dorsal and ventral tissues by neuronal morphology, which together revealed a thorough and prominent cavitation on the hemisected side, resulting in the unilateral sensory and motor defects (Figure 4A). Tissue surrounding the cavity was a little deformed but remained intact, indicating a confined injury by the small incision described in this hemisection model (Christensen et al., 1996). All treatment groups significantly reduced the spinal cavitation area in the injured halve compared with the SCI group (all *P* < 0.001, *n* = 5, Figures 4C,D).

This can also be observed in the lesion area defined by the GFAP-immunoreactive boundary (Figure 4B). Increased immunoreactivity of GFAP, a marker of reactive gliosis, is indicative of neuroinflammation and the formation of astroglial boundary. Here, all treatment groups reduced GFAP immunoreactivities along with cavitation size (Figure 4E). However, the difference between the K and SCI groups (17.8% reduction, *P* = 0.546, K: 2.588 ± 0.4174% vs. SCI: 3.15 ± 0.754%) was not significant. Both groups EE (68% reduction, *P* < 0.001, 1.005 ± 0.2113%) and EEK (52% reduction, *P* = 0.004, 1.462 ± 0.5538%) showed a significantly lower level of astrocytic reactivity than the SCI group (3.15 ± 0.754%) and the K group (EE vs. K: *P* = 0.006; EEK vs. K: *P* = 0.036). Of note, although the EE group exhibited the lowest GFAP immunoreactivity, its lesion size appeared larger than that of the other two treatment groups (38% more than the K group and 63% more than the EEK group). On the contrary, the EEK group had an intermediate GFAP reactivity level but the smallest cavitation among the treatment groups, suggesting that a certain level of astrocytic activity may benefit wound healing.

### Environmental Enrichment Combined With Ketamine Suppresses Perilesional Activation of Neuronal/Microglial *N*-Methyl-D-Aspartate Receptor

**FIGURE 5:**
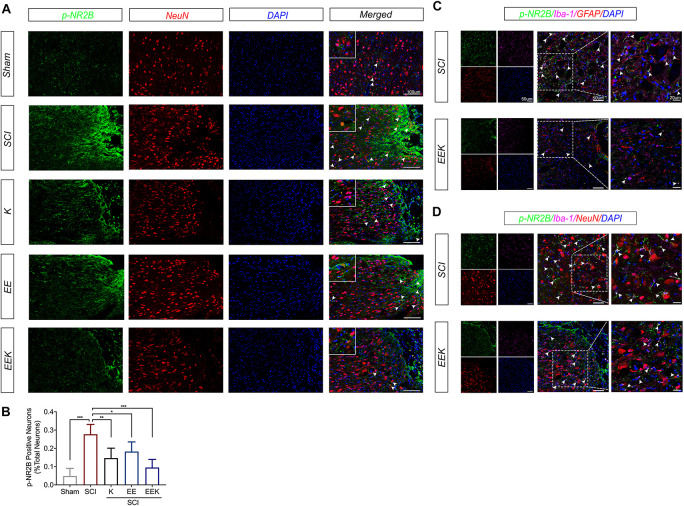
Environmental enrichment (EE) and ketamine (K), especially their combination (EEK), suppressed perilesional activation of neuronal/microglial glutamate receptor after spinal cord injury (SCI). **(A)** Representative images and **(B)** quantification of colocalized phosphorylated *N*-methyl-D-aspartate receptor subtype 2B (p-NR2B) with neuronal marker (NeuN). *n* = 4 in sham, *n* = 6 in SCI, *n* = 4 in K, *n* = 5 in EE, and *n* = 5 in EEK. One-way ANOVA (effect vs. group) followed by Tukey’s *post hoc* test; *B*: *F*(4,19) = 15.72. *P < 0.05, **P < 0.01, ***P < 0.001. Data are presented as mean ± standard deviation (SD). Representative images of triple-labeled p-NR2B with **(C)** microglial (Iba-1) and astrocytic [glial fibrillary acidic protein (GFAP)] markers, or with **(D)** Iba-1 and NeuN. Examples of colocalization are indicated by white arrow heads.

To investigate whether the therapeutic effect of EE and ketamine is associated with neuronal NR2B activation, we double-stained phosphorylated NR2B (p-NR2B) and the neuronal marker (NeuN) on the longitudinal section of the dorsal spinal cord at the injury site. Intense expression of p-NR2B was adjacent to the lesion after SCI, while moderate-to-low expressions were seen in the EE, K, and EEK groups (p-NR2B, Figure 5A). Upon merging with NeuN, colocalization of p-NR2B with a subpopulation of neuron was detected in the forms of overlapping or enwrapping parts of the neuronal cell bodies. This is particularly prominent in the SCI group where p-NR2B-positive neurons spread around and bordering the lesion. In comparison, all treatment groups, especially EEK, showed significantly less p-NR2B-positive neurons (K: *P* = 0.0052, EE: *P* = 0.0372, EEK: *P* < 0.001; *n* = 5, Figure 5B). Of note, the EEK group had scattered dot- or short strand-like expressions of p-NR2B, which resembled those in the sham group. Interestingly, the morphology of p-NR2B expression may also indicate involvement of glia in that EEK may reduce glial activation of NR2B as well.

Hence, we specifically investigated the effect of EEK on any perilesional activation of glial NMDAR. We triple-labeled p-NR2B, microglia, and astrocyte in the SCI and EEK groups. p-NR2B was found to colocalize with microglial cells but not astrocytes in both the SCI and EEK groups (Figure 5C). However, highly expressed p-NR2B overlaid with multiple elongated processes of microglial cells in the SCI group, while a moderate expression of p-NR2B colocalized with a few oval-like microglial cells in the EEK group.

Further, triple staining for p-NR2B, microglia, and neuron revealed active microglial–neuronal interaction in light of activated NMDAR in both cell types in the SCI group (Figure 5D). The morphology of p-NR2B expressions highly resembled active microglia and overlapped with microglial cell markers exhibiting asymmetrically extended processes that closely contacted or ensheathed neurons. In contrast, in EEK, more rounded shapes of microglial cells were observed with partial colocalization. No prominent extensions of microglial processes were recorded.

### Combined Environmental Enrichment and Ketamine Treatment Reduced Glutamatergic Activation in the Lumbar Spinal Cord

**FIGURE 6:**
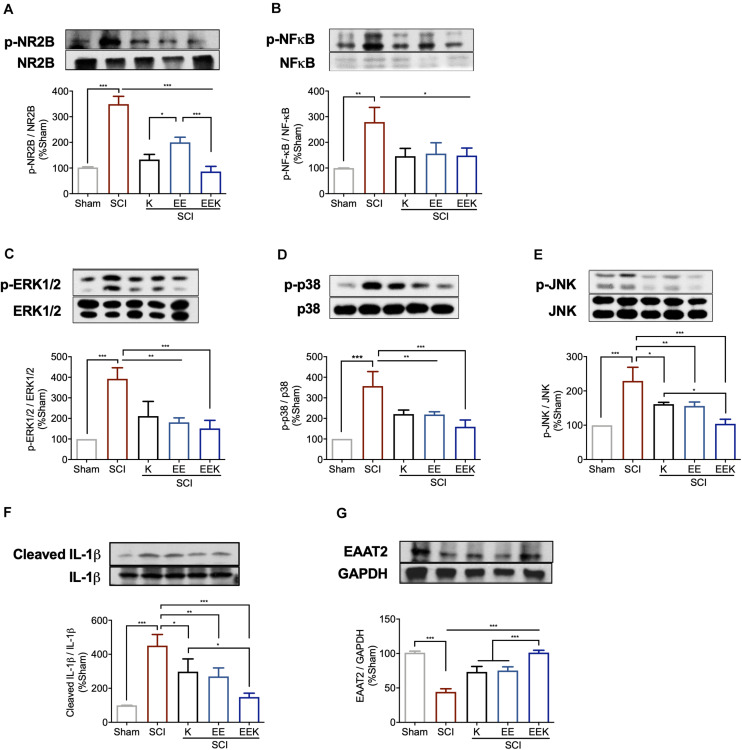
Environmental enrichment (EE) and (K), especially their combination (EEK), reduced glutamatergic regulation-related markers in spinal cord injury (SCI). Representative images of protein expressions of phosphorylated (p) **(A)** NR2B, **(B)** nuclear factor (NF)-κB, **(C)** extracellular signal regulated kinase (ERK) 1/2, **(D)** p38, **(E)** c-Jun N-terminal kinase (JNK), and expressions of **(F)** interleukin (IL)-1β and **(G)** excitatory amino acid transporter (EAAT) 2. *n* = 3/group, one-way ANOVA (effect vs. group) followed by Tukey’s *post hoc* test; **(A)**: *F*(4,10) = 80.35, **(B)**: *F*(4,10) = 9.876, **(C)**: *F*(4,10) = 18.58, **(D)**: *F*(4,10) = 20.83, **(E)**: *F*(4,10) = 21.8, **(F)** *F*(4,10) = 22, **(G)** *F*(4,10) = 66.3. *P < 0.05, **P < 0.01, ***P < 0.001. Data are presented as mean ± standard deviation (SD). Sham, sham-operated group.

We examined the glutamate signaling axis in the lumbar spinal cord, which in part may account for the observed pain-like behaviors caudal to the injury. After SCI, we detected significantly increased activation/phosphorylation of NR2B subunit and its downstream cascade ERK, p38, JNK, and nuclear factor (NF)-κB (all *P* < 0.01, *n* = 3, Figures 6A–F). The protein expression of EAAT2 also decreased significantly (*P* < 0.001, Figure 6G). These alterations were mitigated by all treatments, but to a greater degree by the combined treatment, restoring the dysregulated glutamatergic signaling after SCI.

## Discussion

We demonstrated a novel multi-functional regimen, combining EE housing and ketamine, that effectively improves post-SCI recovery from both motor and nociceptive defects. The beneficial effects of the combined treatment were superior to those of either ketamine or EE alone in locomotor improvement and neuropathic pain relief. In our SCI model, behavioral deficits were observed with marked tissue deterioration, astrogliosis, and activation of neuronal and microglial NR2B subunits of NMDAR at the spinal lesion site. Further, in line with the hindpaw allodynia and hyperalgesia, we found augmented activation of NR2B, MAPK family, NF-κB, and interleukin (IL)-1β, as well as downregulated EAAT2 expression in the lumbar spinal cord. These changes were restored by ketamine, EE, and to a greater extent by their combination as compared with individual treatments, suggesting an additive effect by the joint regimen.

Here, evaluation of EE or ketamine as individual treatment has proven beneficial for post-SCI recovery. Consistent with previous studies (Berrocal et al., 2007; Koopmans et al., 2012), our EE paradigm strengthened locomotion and alleviated neuropathic pain-like behaviors in SCI rats. However, EE treatment alone has a later onset in both the pain-related behavioral and motor function assessments. These data suggest that the impact of EE is slow paced but shows a trend of improvement in that its effect maybe better observed over a long-term scale. This agrees with clinical studies of EE in the rehabilitation unit, which observed better recovery in patients over months (McDonald et al., 2018). To boost its therapeutic efficacy, we adopted the subanesthetic use of ketamine and a multiday-administration scheme, which has been shown to be safe with distinctive efficacy against chronic pain clinically (Goldberg et al., 2005; Amr, 2010). In this study, post-drug analgesic effect of ketamine lasted longer against allodynia (at least 11 days) than against hyperalgesia (at least 7 days), suggesting that the post-drug effect was less effective against noxious stimulus-induced hypersensitivity (hyperalgesia). Although a short-term ketamine administration would be preferable for patients as well as to avoid any potential side effects, the analgesic effect wanes. Therefore, the combination of EE, a long-term feasible application, and ketamine may optimize each other’s therapeutic potential. We showed that the joint regimen generated a profound and prolonged relief of allodynia and hyperalgesia following SCI even after cessation of ketamine. This recovery was seen not only in animals’ pain response but also in their motor function, reflecting a comprehensive advantage of the combined regimen. In the BBB locomotor assessment, the joint treatment achieved full score (score of 21) of motor performance as early as a week after the treatment started. However, the score in rats treated with ketamine or EE alone plateaued at the intermediate-to-late stage of recovery (score of 13 to 18), indicating permanent damage (Basso et al., 1995). Similar to clinical observation, a spontaneous motor recovery after SCI can last for years but remains limited (Fakhoury, 2015). Our finding indicates that the joint treatment of EE and ketamine has a strong potential in locomotor improvement. The burrowing test, which evaluates animal’s well-being and non-stimulus-evoked pain response, also found greater improvement in the EEK group than the individual treatment, which signified an additive effect.

From the morphological study, we found that the joint regimen significantly reduced lesion as shown by quantification of cavity area and astroglial boarder. Interestingly, the EE group has the lowest GFAP immunoreactivity among the three treatment groups but exhibited a slightly larger cavity area than the other two, suggesting certain wound healing by astroglial boundary. Although the glial boundary, also termed glial scar traditionally, was long thought to impede axonal regrowth and neuromotor recovery, it has an intrinsic protective property in response to nerve injury. Recent literature strongly supports the neuroprotective notion of astroglial boarder in that it actually aids axon regeneration (O’Shea et al., 2017; Bradbury and Burnside, 2019). This might explain the moderate astrocytic reactivity and optimal lesion closure in the EEK group, suggesting a balanced act by the joint regimen to optimize tissue recovery. On the other hand, all three treatments reduced neuronal NR2B (p-NR2B) activation at the lesion site, but the joint treatment showed a higher significance in reduction. The sustained overexcitation of neuron in SCI and chronic pain attributes largely to the activation of glutamate NMDAR. Selective inhibition of NR2B-NMDAR produces a significant anti-allodynic effect as well as neuroprotection (Zhuo, 2009; Woolf, 2011). As EE and ketamine both suppressed neuronal NR2B-NMDAR activation, the joint treatment may have combined this effect of the two and produced the better analgesia and neuroprotection in this study. This may also explain the overshoot of PWL in the KEE group after SCI because NMDAR has been indicated in the maintenance of hyperalgesia, suggesting a strong anti-hyperalgesic property of this combined treatment (Skyba et al., 2002). In particular, NR2B-NMDAR is the enriched subtype at extrasynaptic sites, the interactive points of neuron with glia, mediating excitotoxicity and pro-death signaling (Hardingham and Bading, 2010). Potentiated glutamatergic response in the neuron–microglia interaction was reported in neuroinflammation-induced hyperalgesia and neuronal cell death (Kaindl et al., 2012; Sung et al., 2017). To investigate the perilesional NR2B-NMDAR activity in neuron–microglia interaction, we triple-labeled p-NR2B with microglia and neuron markers in the SCI and EEK groups. The morphological comparison of the two revealed less signs of microglia activation and microglial–neuronal contacts in the EEK group, as well as the p-NR2B intensity within them, suggesting that the noted therapeutic advantages may associate with suppressed hyperfunction of extrasynaptic NMDAR. On the other hand, we did not notice colocalization of p-NR2B and astrocyte marker in the spinal sections. Although astrocytic NMDAR has also been mentioned in neuropathogenesis, it was reported in the brain but not the spinal cord (Dzamba et al., 2013).

Besides the injury site, widespread secondary damage following the initial spinal trauma has been observed in the lumbar spinal cord and considered as a molecular basis for below-level neuropathic pain (O’Shea et al., 2017). Glutamate excitotoxicity plays a crucial role in propagating the damage by strengthening excitatory neurotransmission via NMDAR and its downstream MAPK/NF-κB signaling cascade (Willard and Koochekpour, 2013). In addition, nerve injury often stimulates release of IL-1β from microglia, a signature of neuroinflammation. It impairs glutamate clearance by downregulating EAAT2, the major type of glutamate transporter, but also enhances neuronal release of glutamate, aggravating glutamate excitotoxicity (Sung et al., 2017). Together, these events of glutamatergic dysregulation are signature to central sensitization, which underlies neuropathic pain (Woolf, 2011). In line with our behavioral observance, the joint regimen restored these alterations better than did individual treatments. It is worth noting that a novel effect of EE was shown in inhibiting lumbar spinal NR2B-NMDAR, consistent with its suppression on perilesional activation of NR2B, offering an optimal mitigating potential for SCI.

## Conclusion

In summary, we demonstrated a novel multitudinal therapeutic scheme, EE joint with ketamine, which enhanced relief of SCI-induced neuropathic pain and promoted tissue integrity as well as locomotion by targeting the glutamatergic system. Notably, the combined regimen has no adverse effects. But several limitations in this study should be considered. Although the spinal hemisection model has been popular in chronic pain study, this injury rarely occurs in the clinic. Therefore, findings from this study may be replicated in other SCI models, such as contusion injury, to merit further translational investigation. Other potential limitations in the stimulus-evoked pain assessments (von Frey and Hargreaves) should be noted that increased hindpaw withdrawal response could result from hyperreflexia rather than pain after SCI.

While SCI studies that attempt to evaluate multi-approach therapies and the underlying mechanism remain scarce, our data may shed light in this field and encourage future investigation of multi-modal rehabilitations that can minimize secondary damage and maximize residual function.

## Data Availability Statement

The original contributions presented in the study are included in the article/supplementary material, further inquiries can be directed to the corresponding author/s.

## Ethics Statement

The animal study was reviewed and approved by the Committee on the Use of Live Animals in Teaching and Research, The University of Hong Kong.

## Author Contributions

WT and CC designed the study. WT, LS, PG, and HL performed the experiment. WT, LS, HL, PG, and EJ performed the data analysis and interpretation. WT wrote the manuscript. WT, HL, EJ, and CC reviewed and revised the manuscript for final approval. All authors discussed the results and commented on the manuscript. All authors contributed to the article and approved the submitted version.

## Conflict of Interest

The authors declare that the research was conducted in the absence of any commercial or financial relationships that could be construed as a potential conflict of interest.

## References

[B1] Amr Y. M. (2010). Multi-day low dose ketamine infusion as adjuvant to oral gabapentin in spinal cord injury related chronic pain: a prospective, randomized, double blind trial. *Pain Physician* 13 245–249. 20495588

[B2] Andrews N. Legg E. Lisak D. Issop Y. Richardson D. Harper S. (2012). Spontaneous burrowing behaviour in the rat is reduced by peripheral nerve injury or inflammation associated pain. *Eur. J. Pain* 16 485–495. 10.1016/j.ejpain.2011.07.012 22396078

[B3] Basso D. M. Beattie M. S. Bresnahan J. C. (1995). A sensitive and reliable locomotor rating scale for open field testing in rats. *J. Neurotrauma* 12 1–21. 10.1089/neu.1995.12.1 7783230

[B4] Bell J. D. (2017). In vogue: ketamine for neuroprotection in acute neurologic injury. *Anesth. Analg.* 124 1237–1243. 10.1213/ane.0000000000001856 28079589

[B5] Benson C. Paylor J. W. Tenorio G. Winship I. Baker G. Kerr B. J. (2015). Voluntary wheel running delays disease onset and reduces pain hypersensitivity in early experimental autoimmune encephalomyelitis (EAE). *Exp. Neurol.* 271 279–290. 10.1016/j.expneurol.2015.05.017 26033473

[B6] Berrocal Y. Pearse D. D. Singh A. Andrade C. M. Mcbroom J. S. Puentes R. (2007). Social and environmental enrichment improves sensory and motor recovery after severe contusive spinal cord injury in the rat. *J. Neurotrauma* 24 1761–1772. 10.1089/neu.2007.0327 18001204

[B7] Bradbury E. J. Burnside E. R. (2019). Moving beyond the glial scar for spinal cord repair. *Nat. Commun.* 10:3879. 10.1038/s41467-019-11707-7 PMC6713740 31462640

[B8] Christensen M. D. Everhart A. W. Pickelman J. T. Hulsebosch C. E. (1996). Mechanical and thermal allodynia in chronic central pain following spinal cord injury. *Pain* 68 97–107. 10.1016/s0304-3959(96)03224-1 9252004

[B9] Coronel M. F. Labombarda F. Villar M. J. De Nicola A. F. Gonzalez S. L. (2011). Progesterone prevents allodynia after experimental spinal cord injury. *J. Pain* 12 71–83. 10.1016/j.jpain.2010.04.013 20675200

[B10] Costigan M. Scholz J. Woolf C. J. (2009). Neuropathic pain: a maladaptive response of the nervous system to damage. *Annu. Rev. Neurosci.* 32 1–32. 10.1146/annurev.neuro.051508.135531 19400724 PMC2768555

[B11] de la Tremblaye P. B. Bondi C. O. Lajud N. Cheng J. P. Radabaugh H. L. Kline A. E. (2017). Galantamine and environmental enrichment enhance cognitive recovery after experimental traumatic brain injury but do not confer additional benefits when combined. *J. Neurotrauma* 34 1610–1622. 10.1089/neu.2016.4790 27806662 PMC5397275

[B12] de la Tremblaye P. B. Cheng J. P. Bondi C. O. Kline A. E. (2019). Environmental enrichment, alone or in combination with various pharmacotherapies, confers marked benefits after traumatic brain injury. *Neuropharmacology* 145 13–24. 10.1016/j.neuropharm.2018.02.032 29499273

[B13] Deacon R. M. J. (2006). Burrowing in rodents: a sensitive method for detecting behavioral dysfunction. *Nat. Protoc.* 1 118–121. 10.1038/nprot.2006.19 17406222

[B14] Duenas M. Ojeda B. Salazar A. Mico J. A. Failde I. (2016). A review of chronic pain impact on patients, their social environment and the health care system. *J. Pain Res.* 9 457–467. 10.2147/jpr.s105892 27418853 PMC4935027

[B15] Dzamba D. Honsa P. Anderova M. (2013). NMDA receptors in glial cells: pending questions. *Curr. Neuropharmacol.* 11 250–262. 10.2174/1570159x11311030002 24179462 PMC3648778

[B16] Fakhoury M. (2015). Spinal cord injury: overview of experimental approaches used to restore locomotor activity. *Rev. Neurosci.* 26 397–405. 25870961 10.1515/revneuro-2015-0001

[B17] Gabriel A. F. Marcus M. A. E. Honig W. M. M. Helgers N. Joosten E. A. J. (2009). Environmental housing affects the duration of mechanical allodynia and the spinal astroglial activation in a rat model of chronic inflammatory pain. *Brain Res.* 1276 83–90. 10.1016/j.brainres.2009.04.039 19406110

[B18] Goldberg M. E. Domsky R. Scaringe D. Hirsh R. Dotson J. Sharaf I. (2005). Multi-day low dose ketamine infusion for the treatment of complex regional pain syndrome. *Pain Physician* 8 175–179. 16850072

[B19] Hardingham G. E. Bading H. (2010). Synaptic versus extrasynaptic NMDA receptor signalling: implications for neurodegenerative disorders. *Nat. Rev. Neurosci.* 11 682–696. 10.1038/nrn2911 20842175 PMC2948541

[B20] Janssen H. Ada L. Karayanidis F. Drysdale K. Mcelduff P. Pollack M. (2012). Translating the use of an enriched environment poststroke from bench to bedside: study design and protocol used to test the feasibility of environmental enrichment on stroke patients in rehabilitation. *Int. J. Stroke* 7 521–526. 10.1111/j.1747-4949.2011.00727.x 22264219

[B21] Kaindl A. M. Degos V. Peineau S. Gouadon E. Chhor V. Loron G. (2012). Activation of microglial N-methyl-D-aspartate receptors triggers inflammation and neuronal cell death in the developing and mature brain. *Ann. Neurol.* 72 536–549. 10.1002/ana.23626 23109148

[B22] Kentner A. C. Khoury A. Lima Queiroz E. Macrae M. (2016). Environmental enrichment rescues the effects of early life inflammation on markers of synaptic transmission and plasticity. *Brain Behav. Immun.* 57 151–160. 10.1016/j.bbi.2016.03.013 27002704

[B23] Koopmans G. C. Deumens R. Honig W. M. Hamers F. P. Mey J. Van Kleef M. (2012). Functional recovery, serotonergic sprouting, and endogenous progenitor fates in response to delayed environmental enrichment after spinal cord injury. *J. Neurotrauma* 29 514–527. 10.1089/neu.2011.1949 22026514

[B24] McDonald M. W. Hayward K. S. Rosbergen I. C. M. Jeffers M. S. Corbett D. (2018). Is environmental enrichment ready for clinical application in human post-stroke rehabilitation? *Front. Behav. Neurosci.* 12:135. 10.3389/fnbeh.2018.00135 PMC6050361 30050416

[B25] O’Shea T. M. Burda J. E. Sofroniew M. V. (2017). Cell biology of spinal cord injury and repair. *J. Clin. Invest.* 127 3259–3270. 28737515 10.1172/JCI90608 PMC5669582

[B26] Paoletti P. Bellone C. Zhou Q. (2013). NMDA receptor subunit diversity: impact on receptor properties, synaptic plasticity and disease. *Nat. Rev. Neurosci.* 14 383–400. 10.1038/nrn3504 23686171

[B27] Pourmand A. Mazer-Amirshahi M. Royall C. Alhawas R. Shesser R. (2017). Low dose ketamine use in the emergency department, a new direction in pain management. *Am. J. Emerg. Med.* 35 918–921. 10.1016/j.ajem.2017.03.005 28285863

[B28] Ramer L. M. Ramer M. S. Bradbury E. J. (2014). Restoring function after spinal cord injury: towards clinical translation of experimental strategies. *Lancet Neurol.* 13 1241–1256. 10.1016/s1474-4422(14)70144-9 25453463

[B29] Rutten K. Schiene K. Robens A. Leipelt A. Pasqualon T. Read S. J. (2014). Burrowing as a non-reflex behavioural readout for analgesic action in a rat model of sub-chronic knee joint inflammation. *Eur. J. Pain* 18 204–212. 10.1002/j.1532-2149.2013.00358.x 23853119

[B30] Schwartzman R. J. Alexander G. M. Grothusen J. R. Paylor T. Reichenberger E. Perreault M. (2009). Outpatient intravenous ketamine for the treatment of complex regional pain syndrome: a double-blind placebo controlled study. *Pain* 147 107–115. 10.1016/j.pain.2009.08.015 19783371

[B31] Shi X. Guo T. Z. Li W. Sahbaie P. Rice K. C. Sulima A. (2018). Exercise reverses nociceptive sensitization, upregulated neuropeptide signaling, inflammatory changes, anxiety, and memory impairment in a mouse tibia fracture model. *Anesthesiology* 129 557–575. 10.1097/aln.0000000000002332 29994924 PMC6092202

[B32] Siddall P. J. Mcclelland J. M. Rutkowski S. B. Cousins M. J. (2003). A longitudinal study of the prevalence and characteristics of pain in the first 5 years following spinal cord injury. *Pain* 103 249–257. 10.1016/s0304-3959(02)00452-9 12791431

[B33] Skyba D. A. King E. W. Sluka K. A. (2002). Effects of NMDA and non-NMDA ionotropic glutamate receptor antagonists on the development and maintenance of hyperalgesia induced by repeated intramuscular injection of acidic saline. *Pain* 98 69–78. 10.1016/s0304-3959(01)00471-7 12098618

[B34] Sleigh J. Harvey M. Voss L. Denny B. (2014). Ketamine – more mechanisms of action than just NMDA blockade. *Trends Anaesth. Crit. Care* 4 76–81. 10.1016/j.tacc.2014.03.002

[B35] Stagg N. J. Mata H. P. Ibrahim M. M. Henriksen E. J. Porreca F. Vanderah T. W. (2011). Regular exercise reverses sensory hypersensitivity in a rat neuropathic pain model: role of endogenous opioids. *Anesthesiology* 114 940–948. 10.1097/aln.0b013e318210f880 21386701 PMC6345518

[B36] Sun L. Li H. Tai L. W. Gu P. Cheung C. W. (2018). Adiponectin regulates thermal nociception in a mouse model of neuropathic pain. *Br. J. Anaesth.* 120 1356–1367. 10.1016/j.bja.2018.01.016 29793601

[B37] Sung C.-S. Wen Z.-H. Feng C.-W. Chen C.-H. Huang S.-Y. Chen N.-F. (2017). Potentiation of spinal glutamatergic response in the neuron-glia interactions underlies the intrathecal IL-1β-induced thermal hyperalgesia in rats. *CNS Neurosci. Ther.* 23 580–589. 10.1111/cns.12705 28544775 PMC6492640

[B38] Tai L. W. Pan Z. Sun L. Li H. Gu P. Wong S. S. C. (2018a). Suppression of Pax2 attenuates allodynia and hyperalgesia through ET-1-ETAR-NFAT5 signaling in a rat model of neuropathic pain. *Neuroscience* 384 139–151. 10.1016/j.neuroscience.2018.05.024 29847776

[B39] Tai L. W. Yeung S. C. Cheung C. W. (2018b). Enriched environment and effects on neuropathic pain: experimental findings and mechanisms. *Pain Pract.* 18 1068–1082. 10.1111/papr.12706 29722923

[B40] Willard S. S. Koochekpour S. (2013). Glutamate, glutamate receptors, and downstream signaling pathways. *Int. J. Biol. Sci.* 9 948–959. 10.7150/ijbs.6426 24155668 PMC3805900

[B41] Woolf C. J. (2011). Central sensitization: implications for the diagnosis and treatment of pain. *Pain* 152 S2–S15. 20961685 10.1016/j.pain.2010.09.030 PMC3268359

[B42] Zhuo M. (2009). Plasticity of NMDA receptor NR2B subunit in memory and chronic pain. *Mol. Brain* 2:4. 10.1186/1756-6606-2-4 19192303 PMC2644299

